# Discovery of continental-scale travelling waves and lagged synchrony in geometrid moth outbreaks prompt a re-evaluation of mountain birch/geometrid studies

**DOI:** 10.12688/f1000research.2-128.v2

**Published:** 2013-09-06

**Authors:** Olle Tenow

**Affiliations:** 1 Department of Ecology, Swedish University of Agricultural Sciences, Uppsala, SE-750 07, Sweden

**Keywords:** Operophtera brumata; Epirrita autumnata, defoliation, Scandinavia, population dynamics

## Abstract

The spatio-temporal dynamics of populations of two 9-10 year cyclic-outbreaking geometrids, *Operophtera brumata* and *Epirrita autumnata* in mountain birch forests in northern Fennoscandia, have been studied since the 1970´s by a Swedish-Norwegian research team and, during the last decade, by Norwegian and Finnish research teams. Some of the early results have been challenged by the Norwegian team. To examine the base for disagreements, five of the papers published by the Norwegian team (2004-2011) are reviewed. It is found that conclusions in these papers are questionable or data could not be interpreted fully because two decisive traits in the spatio-temporal behaviour of outbreaks of the two species were not considered.

## Introduction

Two recent observations of the spatio-temporal outbreak pattern of some extensively studied geometrid moth species necessitate a re-evaluation of previously published findings. Firstly, it has been demonstrated that defoliating outbreaks on deciduous trees of the winter moth ( *Operophtera brumata* L.) and associated geometrids every 9–10 years travel as a wave across Europe from east to west with the front of the wave stretching from high to low latitudes ^1^. Secondly, long-term studies on the temporal behaviour of *Epirrita autumnata*, an associated geometrid, and *O. brumata* in the mountain chain of Scandinavia (the Scandes) and northern Finland show that outbreaks of *O. brumata* have been synchronized with those of *E. autumnata* with a time lag of about 1–2 years ^2– 4^.

Temporal dynamics of populations of cyclic geometrids have been studied in some parts of central Europe (e.g. Raimondo *et al.* 2004 ^5^; Glavendekić 2002 ^6^; Kula 2008 ^7^). However, research on spatio-temporal behaviour seems to have been undertaken only in Fennoscandia (Norway, Sweden and Finland), i.e., on *O. brumata*, *E. autumnata* and *Agriopis aurantiaria* on birch ( *Betula pubescens* and *B. p. czerepanovii*=mountain birch). These studies started with a historical survey of outbreaks on birch of *E. autumnata* and *O. brumata* that covered the years 1862–1968 in the geographical area of Fennoscandia ^8^. It revealed that 12 outbreak periods had occurred during the surveyed period, with an average interval of 9–10 years. The survey was followed-up for the years 1969–2001 ^9^, adding three more outbreak periods, this time with quantitative data (including for *A. aurantiaria*) for the period 1990–2001 ^3^. In these studies, it was found that outbreaks sometimes occurred contemporaneously along the Scandes and sometimes moved as a wave from north to south along the Scandes or from south to north. Although true within the scope of these studies, it has now been demonstrated that the patterns described are illusions caused by continental-scale outbreak waves passing the Scandes either in parallel or obliquely from north or south ^1^. Another study, also restricted to Fennoscandia, showed that waves of *E. autumnata* outbreaks during the four outbreak periods from 1970–2004 have, on average, travelled across Fennoscandia from about NE/E to SW/W ^1^. This aligns with the results in Tenow *et al.* (2013) ^1^.

In Tenow *et al.* (2013) ^1^, it is argued that local and even regional population dynamics of a species cannot be properly understood if large-scale waves in cyclic populations occur without them being recognized. The prime examples of such misinterpretations on a regional scale are the descriptions of outbreak waves in the above-mentioned papers ^8, 9^. The purpose of this paper is to expose further examples of similar shortcomings.

### Commentary on five papers regarding the local and regional spatio-temporal population behaviour of *O. brumata* and *E. autumnata*

In 1999, a Norwegian project was launched to cover one cycle of the population ecology of *O. brumata* and *E. autumnata* in northern Fennoscandia. The project has hitherto (2013) produced 22 papers ( http://www.birchmoth.com/) of which nine focus on local and regional spatio-temporal population behaviour of mainly *O. brumata*. The results and conclusions in five of them are relevant in the present context. These papers are Ims *et al.* (2004) ^11^, Hagen *et al.* (2008) ^12^, Jepsen *et al.* (2009a) ^13^, Jepsen *et al.* (2009b) ^14^ and Jepsen *et al.* (2011) ^15^. The papers are interconnected, and to a large extent written by the same authors, and are therefore considered together. They are commented on in order of publication year.

**Figure 1.**
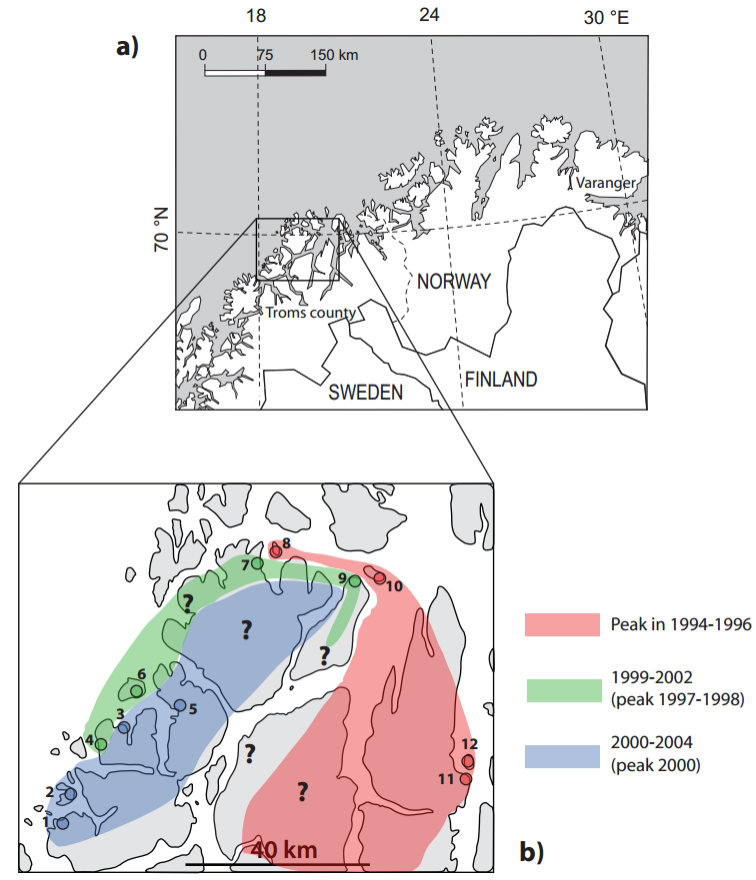
**a**) Map of northern Fennoscandia where defoliations of birch forests during outbreaks of *E. autumnata* and *O. brumata* in 2000–2008 were mapped from satellite imagery (Jepsen *et al.* 2009a ^13^; 2009b ^14^). Framed area marks the area for intensive studies of *O. brumata* occurrence in 1999–2009 (Ims *et al.* 2004 ^11^; Hagen *et al.* 2008 ^12^; Jepsen *et al.* 2011 ^15^). **b**) Map of intensive study area with study sites and indicated movement directions of the wave of the 1990s *O. brumata* occurrence (Ims *et al.* 2004 ^11^; Hagen *et al.* 2008 ^12^; Jepsen *et al.* 2011 ^15^). Pink = the wave has passed the sites and populations are in the low phase; green = crash phase; blue = peak and crash phases. Island sites: 12 (Årøy), 10 (Karlsøy), 8 (Dåvøy), 6 (Vengsøy), 4 (Rekvik), 2 (Tussøy). Mainland sites: 11 (Lyngen), 9 (Reinøy), 7 (Dåfjord), 5 (Skulsfjord), 3 (Tromvik), 1 (Sandvik). Numbers of sites are from Jepsen *et al.* (2011, Figure 3) ^15^; corresponding names from Ims *et al.* (2004, Figure 1) ^11^.

For information on the investigation region, area and sites, see maps, Figure 1, modified from Tenow *et al.* (2007, Figure 3) ^3^ and Jepsen *et al.* (2011, Figure 3) ^15^.

Ims *et al.* (2004) Do sub-Arctic winter moth populations in coastal birch forest exhibit spatially synchronous dynamics? *Journal of Animal Ecology* 73: 1129–1136 ^11^.

This paper deals with the spatio-temporal behaviour of *O. brumata* populations in north-western-most Norway (Troms County; Figure 1a) in 1999–2002 ( Figure 1 in the paper; cf. Jepsen *et al.* 2011 below ^15^). *E. autumnata* was omitted from the study because of insufficient numbers in this coastal area. In an early study ^8^ based on historical documentation, it was suggested that climatic forcing (the Moran effect) may have synchronized the 9–10-year cyclic outbreaks of *O. brumata* (and *E. autumnata*) occurring in the Scandes and northern Finland. The actual study raised the question as to whether this suggestion withstands closer scrutiny in terms of more detailed quantitative data. Commenting on the short duration of the study, it is said

“… *although studies of dynamics of cyclic populations for many purposes need to span a longer time period than the time period of a cycle (i.e., 10 years for the focal moth species) the degree of synchronous dynamics can be evaluated from more short termed data (.....)"*, and, *"for cyclic populations, identifying and comparing the phase among sites at any given time would be sufficient to verify the prevalence of synchrony. Here we use the latter approach on our 4-year data set…*”.

The female *O. brumata* moth has stunted wings and cannot fly. Physical barriers, such as sea water between mainland and islands at the coast, may therefore prevent the spread of females and isolate island populations. On the understanding that the climate is identical, differing population dynamics on the mainland and nearby islands should imply the disruption of the Moran effect. Six pairs of sites were selected from east to west in the north-western coastal part of Troms, each with one site on the mainland (or a large island) and one on a medium-sized island, separated by >1.5 km of open sea ( Figure 1b). At each site, the number of caterpillars per sampled twig at a number of stations up-slope was recorded over four years (1999–2002).

It was found that population densities were low on all islands, except for one (site 2, Tussøy), and that populations there were maximally out of phase with peaking populations on the mainland, or populations that had recently peaked, except for one (site 1, Sandvik). The authors concluded that

“… *the distinct asynchrony between adjacent sites (<10 km apart) clearly belonging to the same climatic domain rules out the possibility that climate or, for that matter any other supposedly large-scale phenomenon (.....) could be responsible for the population phase differences*”.

Thus, the notion of a large-scale spatial synchrony due to a Moran effect was rejected, at least for coastal birch forest.

Later, it was shown ^3^ that a large-scale *E. autumnata/O. brumata* outbreak wave arrived at northern Fennoscandia from the east in 1991–1992. The wave travelled westward across the region and reached the eastern part of Troms in 1994–1995, and finally the west coast of Troms in about 2000 where the mainland/island study was performed. It was stated in Tenow *et al.* (2007) ^3^ that this wave must be considered when the results of that study are to be interpreted.

Since then, Jepsen *et al.* (2011) ^15^ have added seven more years to the data set, which now comprises the years 1999–2009. This offers an opportunity to re-evaluate the 2004 results. The low density island sites, seen in Figure 1 in the paper, may be subdivided into three groups from east to west within the investigation area (cf. enlargement of population curves for 1999–2002 in Jepsen *et al.* 2011, Figure 3, and comments to that paper below ^15^): one group consisting of the easternmost site and the two northernmost sites that experienced an evident low during the study time (1999–2002) ( Figure 1b: sites 8, 10, 12) and one group of the next two island sites to the south-west that underwent the greatest crash phase during the same period (sites 4, 6). Finally, the south-western-most island site (2) exhibited a full peak and a crash. The mainland sites may also be divided into three groups; the easternmost site with an evident low during the study (11), the two northernmost sites with a crash (7, 9; although site 9 shows a deviating population curve), and the three south-western-most sites with a full peak and a crash (1, 3, 5). This demonstrates that the wave of the 1990s, which arrived to eastern Troms in 1994–1995 and then continued westward, had already passed across the northern part of the investigation area before the study, i.e., about the middle of the 1990s. Then, in 1999–2002, the wave was recorded at the south-western island sites in its crash phase and finally, in 2000–2004, it was caught up lingering at the south-western-most mainland sites in its peak and crash phases. Hence, by first reaching the island sites in the north in the direction E-W, then the island sites along the west coast in the direction N-S, and finally the mainland sites inside the islands, it behaved like a wave "breaking" successively over the mainland part of the investigation area ( Figure 1b). Of the six pairs of sites, only one (9, 10) indicates populations that may have been maximally out of phase-one branch of the wave passed south of the investigation area already in 1994–1995 ^3^.

In conclusion, there were no distinct, maximally out of phase of population dynamics between sites on mainland and nearby islands. Hence, the Moran effect cannot be discarded. Instead, there was a continuous progression over the investigation area of the large-scale wave of the 1990s, first via the island sites then to the mainland sites. Therefore, at any given time, populations separated in space in the direction of the wave had been in continuous change in larval density. Furthermore, the continuation of the study (cf. Jepsen *et al.* 2011 ^15^) clearly demonstrates that a four-year study in this case was not sufficient to allow a correct interpretation.

Hagen *et al.* (2008) Anisotropic patterned population synchrony in climatic gradients indicates nonlinear climatic forcing. *Proceedings of the Royal Society* B 275: 1509–1515 ^12^.

The paper gives detailed data on spatio-temporal changes in larval abundance of *O. brumata* and *E. autumnata* in vertical and horizontal directions on a coastal mountain slope ( Figure 1b: site 9, Reinøya) in 2001–2007. It is assumed that the air temperature gradients decrease linearly with increasing altitude whereas the temperature is approximately the same in a horizontal direction at each altitude. It is predicted that the varying sensitivity of different insect life stages to climate can cause a non-linear forcing on population synchrony along altitudinal air temperature gradients on slopes. If there is a nonlinear forcing in this case, a synchronisation caused by a Moran effect could be rejected. It is stated that the `global spline correlogram´, applied for each species, was based on all population time series (i.e., 2001–2007). In the beginning of the results section, the authors stated:

“ *The 7-year time series of both species exhibit sufficient temporal variation to provide an useful basis for investigating patterns of population synchrony (….). That is, all populations had crashed at the end of the study, enabling us to highlight synchrony of the crash phase of the population cycle*”.

It is shown that *E. autumnata* went through all stages of a regular cycle (increase, peak, and crash during 5 years, followed by a low) during the 7 years. The changes of the *O. brumata* population showed more variation but the overall trend was less steep and often lagged behind *E. autumnata*. There is no mention of cycle phases other than the crash phase ( *O. brumata*), nor mention of successive population cycles or of outbreak waves (cf. Tenow 1972 ^8^; Tenow *et al.* 2007 ^3^) only of “more complex spatial patterns”. Directional (anisotropic) correlograms for *O. brumata* showed that the synchrony declined steeply in altitudinal direction but not in the horizontal direction of the slope. *E. autumnata* did not exhibit any anisotropy in either direction.

These results can be compared with Schott *et al.* (2010) ^16^, a study based on the same data. That study clearly points out a crash phase for *O. brumata*, for the cycle of the 1990s, in addition to that of the first decade of the twenty-first century. The crash of the 1990s occurred in 2001 or 2000–2001, followed by a population low in 2002 (Schott *et al.* 2010, Figure 3 ^16^, most distinct at higher altitudes). The same low is visible, although faint, for the same site in Jepsen *et al.* (2011; Figure 3 ^15^). For *E. autumnata*, the crash phase of the cycle of the 1990s had occurred before the start of the project. The low occurred in 2001 or 2000–2001, i.e., 1–2 years ahead of *O. brumata*. Evidently, the low for each species separated the cycle/wave of the 1990s (cf. Ims *et al.* 2004 above ^11^) from the following one in the 2000s (cf. Jepsen *et al.* 2011 below ^15^). For *O. brumata*, the peak of the latter cycle occurred in 2004–2005 at the two highest altitudes, followed by a crash at all altitudes in 2006–2007. For *E. autumnata*, the peak of the same cycle occurred contemporaneously at all altitudes in 2002–2003, two years ahead of *O. brumata* at the two highest altitudes (Schott *et al.* 2010, Figure 3 ^16^). The crash followed in 2005–2006, also at all altitudes.

The *O. brumata* low in 2002, shown in Schott *et al.* (2010, Figure 3 ^16^), means that 2002 was a year of transition between the *O. brumata* cycle of 1990s and that of the 2000s. In the analysis of the spatially directed (anisotropic) synchrony patterns, this low was not detected although visible with an expected time lag after *E. autumnata* in Figure 4 of the paper (cf. Schott *et al.* 2010, Figure 3 ^16^), and the 7-year time series for *O. brumata* seems to have been treated as one single long crash phase. It is important here to point out that no specific cycle (increase, peak and crash) lasts 7 years, not to mention the crash phase, which usually is the most rapid of the three phases. As a consequence, two successive *O. brumata* cycles became involved in the analysis, the crash in 2001 of the cycle of the 1990s, the low in 2002 and the full cycle of the 2000s. According to Figure 3 in Schott *et al.* (2010) ^16^, populations at the same altitudes (240, 170 and 100 m above sea level, respectively) were much less synchronized during the crash of the cycle of the 1990s than during phases of the cycle of the 2000s, including the crash. Despite the fact that these fluctuations constitute a minor part in the analysis, they may have created undue variation. In addition, to be comparable, the analysis for the two geometrids should probably have covered the same phases of the outbreak cycle (increase, peak, crash), or the years 2002–2004 (2005) for *E. autumnata* and, with the time lag, the years 2003–2005 (2006) for *O. brumata*. It is obvious from Figure 4 in the paper that the spatio-temporal population behaviour during the same phases was similar for the two species. These circumstances cast doubt on the claim that there was an “anisotropic patterned population synchrony in climatic gradients” for *O. brumata*.

The “target” concept is mentioned in the paper; implying that the target, the Moran effect, will be aimed at with precision and accuracy. However, the precision of advanced statistical methods is in vain when accuracy is poor, as can be suspected in this case. The study did not account for the cycles in two successive outbreak waves that passed the site. This makes it open to questions. Does this omission imply that the analysis of the *O. brumata* data became biased and that the result therefore is unreliable?

Jepsen *et al.* (2009a) Monitoring the spatio-temporal dynamics of geometrid moth outbreaks in birch forest using MODIS-NDVI data. *Remote Sensing of Environment* 113: 1939–1947 ^13^.

This study monitored the defoliations of mountain birch forests across northern Fennoscandia ( Figure 1a) in 2000–2008. This was achieved by satellite imagery and the different contributions from the two geometrids *E. autumnata* and *O. brumata* were not specified. One result identified was that the defoliation had spread north-eastward along the mountain chain. Thereby, the conclusion in Tenow *et al.* (2007) ^3^ is challenged namely that outbreaks, like that during the preceding period 1990–1999, obey a consistent pattern by moving in a wave-like fashion broadly from east to west. Instead, it is stated that

“ *Even without a formal analysis of the spatial dynamics of defoliation, our data indicate that this is not so. Future analysis of the spatio-temporal dynamics of defoliation patterns will substantiate this*”.

In any case, here the existence of unidirectional outbreak movements in northern Fennoscandia seems to be accepted (cf. below).

The surveyed region stretches from Varangerfjord in north-eastern-most Norway to the outer coast of Troms County in north-western Norway, a distance of about 400 km ( Figure 1a). Visible defoliations appeared first at the inner part of Varangerfjord in 2002 where they peaked in 2003. In 2004, widespread defoliations could be seen in the middle and the south-western-most parts of the surveyed region (south-west Troms and adjoining parts of Sweden and Finland, Figure 1a). In 2005, they faded away farthest to the southwest while expanding in the middle part. In 2005, defoliations were again visible at Varangerfjord. They peaked in 2006–2007 and ceased in 2008 (Figure 7 in the paper).

It is known that the defoliations at Varangerfjord in 2002–2003 were caused by *E. autumnata* (Klemola, T. *et al.* 2008, Figure 1 ^4^). It is also known that the 2004–2005 defoliations in the middle, inner parts, e.g. Kilpisjärvi-Enontekiö ( Forststyrelsen, Finland; Finlands Natur 4/5/2005) and Karasjok-Kautokeino areas in Norway and Finland ( Skogskader på Internet 22/6/2005) and in the inner, south-western-most part, e.g. in Dividalen in Norway ( Skogskader på Internet 6/7/2004) and the Lake Torneträsk-Kiruna area in Sweden ( Entomologiska Sällskapet i Lund 24/9/2004; Dagens Nyheter 26/7/2004) were caused by *E. autumnata*. Finally, there were again defoliations at Varangerfjord in 2005–2007, however, this time by *O. brumata* ^4^. It is stated that

“ *Where the two species occur sympatrically within their outbreak range, there is a partial interspecific synchrony in the timing of the outbreaks, but with winter moth often lagging 1–2 years behind autumnal moth*....”.

If this well-known 1–2 year lag (e.g. Hagen 2007 ^17^; Tenow *et al.* 2007 ^3^; Klemola *et al.* 2008 ^4^) is applied to the satellite mapping (in addition to the acceptance of waves), it is evident that there was also a lagged defoliation pattern in this case, i.e., defoliations by *E. autumnata* in 2002–2003 at Varangerfjord and then by *O. brumata* in 2005–2007 in the same area. In the meantime, the *E. autumnata* defoliations had spread westward to the middle and south-western parts in 2004. In 2005, the *E. autumnata* defoliations faded away in the south-western-most part while expanding in the middle part of the region. Therefore, when the species and time lag were not recognized, the pattern seen by satellite in 2004–2007 was erroneously interpreted as outbreaks moving from the south-western part in 2004 via the middle part in 2005 to Varangerfjord in 2005–2007, that is from SW to NE. As has been shown, the consistent pattern of outbreak movements is the reverse; that outbreak waves travel broadly from east to west in northern Fennoscandia (1, 3, 10). In accordance with a broadly E-W wave, *O. brumata* peaked at outbreak levels at inner coast sites of Troms in 2006 ^17^ and at low densities further west at outer coast sites in 2006–2008 (Jepsen *et al.* 2011, Figure 3, site 1, 3, 5; cf. Figure 1b ^15^), with a subsequent outbreak here in 2007–2008 (Skogfjord, Ringvassøya; cf. also Jepsen *et al.* 2009b below ^14^), i.e., with a delay of about 1 and 2 years, respectively, compared with Varangerfjord.

It can be concluded that the outbreak wave of the 2000s travelled broadly E-W over northern Fennoscandia as waves have done as far back in time as can be surveyed and not occasionally in the opposite direction as stated in the paper.

Jepsen *et al.* (2009b) Phase-dependent outbreak dynamics of geometrid moth linked to host plant phenology. *Proceedings of the Royal Society* B 276: 4119–4128 ^14^.

This study is based on the same 2000–2008 satellite imagery mapping of defoliations across northern Fennoscandia ( Figure 1a) as used by Jepsen *et al.* (2009a; above) ^13^. The satellite mapping did not discriminate between *E. autumnata* and *O. brumata* defoliations because, again, their different contributions could not be separated. The authors applied two analytical approaches to the spatio-temporal defoliation data. The first approach was focused on population synchrony, namely to assess how synchrony depends on distance. Synchrony can take the form of phase-dependent synchrony, meaning that the degree of synchrony may differ between the increase, peak and crash phases (here evidently implying that phases for the two species coincided). The second approach focused on the rate of spread of defoliation. It was found that defoliation could be divided in three phases. Thus, the analyses comprised estimation of the area of defoliated forest during each of the *incipient* (2000–2002), *epidemic* (2003–2005) and *crash* (2006–2008) *phases* (Figure 2 in the paper). The analyses revealed that the *incipient phase* was characterized by high regional synchrony and long defoliation spread distances per year, whereas the *epidemic* and *crash phases* were characterized by much lower regional synchrony and a much shorter spread of defoliation. This suggests two independent spread-steps, one long-range process and one very short-range diffusion-like process. As for defoliation, the *incipient phase* of the outbreak showed a higher regional synchrony in the onset of plant growth (budburst) than the *later phases* of the outbreak.

There is no discussion about potential outbreak waves crossing the region (cf. Klemola. 2006 ^10^; Tenow *et al.* 2007 ^3^). It is assumed (in the Introduction) that the spatio-temporal synchrony of large-scale outbreaks may be explained by a putative regionalized Moran effect, e.g. a match/mismatch between moth and host phonologies

“ *Such a phenological mismatch-driven Moran effect could also be responsible for the more complex dynamics recently reported for birch forest moths (Klemola et al. 2006; Tenow et al. 2007)*”.

We now know that geometrid outbreak wave travelled from south-eastern Europe to the Atlantic coast in the 2000s and in the 1990s (as well as in earlier decades ^1^). It is evident from comments to papers above that both waves crossed the region. During the investigation period (2000–2008), the wave of the 1990s still lingered during 2000–2004 in the coastal area farthest to the west ( Figure 1b: sites 1–3, 5; Jepsen *et al.* 2011, Figure 3 ^15^; cf. below). It also embraced the low between the two waves, which should have been in 2000–2002, as a mean for this particular region (Figure 2 a in Tenow *et al.* 2013 ^1^), that is, during the *incipient phase*. At the end of a low, local populations become released from delayed density-dependent constraints. In older literature, this phase is named the latent phase, with populations still low but prone to increase, for the geometrids according to their 9–10-year cyclicity. The average travel speed of the wave during the 2000s can be approximated to about 300 km/year (Figure 2 a in Tenow *et al.* 2013 ^1^). When the low between the two waves travelled westward at about 300 km year by year, the populations passed by the low should have been in the latent/increase phase at that point. This should apply for most of the populations within the approximately 400 km wide region mapped during the 2000–2002 low (cf. Jepsen *et al.* 2009a above ^13^).

The average speed of about 300 km/year implies that the wave should have travelled the distance from Varangerfjord to the coast of Troms in about 2 years, allowing for delay in some coastal areas (Figure 3 in Tenow *et al.* 2007 ^3^; Figure 4 in Tenow *et al.* 2013 ^1^). This is in accordance with the time lapse of about 2 years between *E. autumnata* outbreaks at Varangerfjord in 2002–2003 and in the south-western most part of the region in 2004–2005 (Jepsen *et al.* 2009a above ^13^), a distance of about 400 km, and of about 2 years between *O. brumata* outbreaks at Varangerfjord in 2005–2007 and at Skogfjord at the coast in 2007–2008 (Jepsen *et al.* 2009a above ^13^).

A time lag between the defoliations is not discussed despite being acknowledged in previous papers ^12, 17^. Instead, it is stated that *E. autumnata* and *O. brumata*

“.... *exhibit largely synchronous dynamics, with winter moth dominating at termination of the outbreaks*...”.

The lag between species was distinct for the Varangerfjord area to the east (high populations ^4^). Also, in the western part ( Figure 1b), a time lag occurred between the two species (low populations) at site 11 (Lyngenfjord) ^18^ and was repeated one year later at site 9 (Reinøya) to the northwest ^16^.

Because of the wave and the lag, the phase-dependent outbreak dynamics depicted was in reality two separate dynamics, one for each outbreak species. The *incipient phase* (2000–2002) that occurred between the two waves should mainly have consisted of latent and slowly increasing populations of *E. autumnata* over part of the region first, followed later by *O. brumata*. Released in 2000–2001, the *E. autumnata* populations at Varangerfjord peaked in 2002–2003, partly within the *incipient phase*, with increasing areas being defoliated without any significant participation of *O. brumata* ^4^. They then crashed in 2004–2005 during the *epidemic phase*. The *O. brumata* defoliations did not begin to increase in the Varangerfjord area until 2003–2004 where they peaked in 2005–2006 ^4^ at the transition between the *epidemic* and *crash phases*, thus dominating in defoliated areas during the first year of the *crash phase* (cf. Figure 7 in Jepsen *et al.* 2009a ^13^). Finally, *O. brumata* crashed in 2007–2008 ^4^ (Figure 7 in Jepsen *et al.* 2009a ^13^) at the end of the *crash phase*. As the wave of outbreaks proceeded westward, *E. autumnata* peaked over most of the region in 2004–2005 and thereby completely dominated in defoliated areas during the *epidemic phase*. On the other hand, *O. brumata* came to peak at the west coast of Troms in 2006 ^17^ and 2007–2008 (Jepsen *et al.* 2011, Figure 3 ^15^) during the *crash phase*. In this area, the satellite did not record any extensive defoliation despite severe outbreaks. The topography is steep and the forest is fragmented. Because of this, the area of *O. brumata* defoliation is most likely to have been underestimated in the western coastal region compared with the area in the Varanger region with its smoother topography and more continuous forests ^13^. This is supported by reports on large *O. brumata* outbreaks across northern Norway in 2006 ^17^, i.e., in areas that were closer to the coast than the *E. autumnata* outbreaks (cf. Jepsen *et al.* 2008 ^19^). When summarising the time scales, it is obvious that *E. autumnata* dominated defoliation that moved progressively from east to west during the *incipient* and *epidemic phases* (2002–2005) and *O. brumata*, with some overlap, in a similar way during the *crash phase* (2005–2008), all according to the time lag between the species. Thus, neither dynamic fitted the applied presumed periods of the phase-dependent synchrony.

According to the definition of a wave, defoliations that were widely separated in space can hardly have been contemporaneous during the *incipient*, *epidemic* and *crash* phases. In addition, these phases are split in one and the same area due to the time lag between species. Therefore, it cannot be claimed that the spread of defoliation was determined by a *stationary*, step-wise process, one incipient long-range step and one later short-range.

The onset of plant growth (budburst) had a phase-dependent pattern of spatial synchrony during the *incipient phase* that was similar to that in the occurrence of defoliation (above; although with a much higher level of spatial synchrony for the former). This suggested to the authors that spring phenology plays a decisive role in the synchrony of moth outbreaks, i.e., an indirect Moran effect. However, contrary to defoliation, spring and budburst do not arrive to the region as a wave moving broadly from east to west. In line with a much higher level of spatial synchrony for budburst than for defoliation (above), budburst may occur over large areas at the same time and, probably, does not have much to do with the degree of defoliation (cf. Bylund 1999 ^20^; Nilssen *et al.* 2007 ^9^). Defoliation events in northern Fennoscandia (as well as in Fennoscandia as a whole ^8^), are cyclic and tightly connected to the outbreak waves that pass broadly from east to west approximately every 9–10 years ^1, 3, 10^. Why these waves travel as they do and if/how they force cyclicity upon local populations is not known. What can be said is that there is no known direct or indirect effect of climate forcing on any match/mismatch of plant/insect synchrony that, firstly, can cause a 9–10-year cyclicity locally and, secondly, travels across Europe from east to west every 9–10 years ^1^. In addition, in northern Fennoscandia such an effect should act twice, first on *E. autumnata* and then on *O. brumata*, with a lag of 1–2 years. Should the match/mismatch apply only to *E. autumnata* or to both species?

In conclusion, the division of outbreak dynamics in phases with different synchrony and spread speed must be questioned. Instead, it is the wave and the time lag that are important. Furthermore, contrary to what is suggested, there can be no climate-mediated Moran effect that drives the 9–10 year passages of continental travelling waves over the region and/or drives the local 9–10-year population cycles.

Jepsen *et al.* (2011) Rapid northwards expansion of forest insect pest attributed to spring phenology matching with sub-Arctic birch. *Global Change Biology* 17: 2071–2083 ^15^.

This paper surveys the time period 1999–2009 and offers interesting quantitative data on the occurrence of *O. brumata* at the same 6 pairs of sites in the western, coastal part of northern Fennoscandia as mentioned above ( Figure 1b; Ims *et al.* 2004 above ^11^). The main purpose of the study was to describe and explain a recent invasion of *A. aurantiaria* in the area; outbreak waves were not considered.

Taken together, comments on the papers above reveal that waves of *O. brumata* population peaks have travelled broadly from east to west in northern Fennoscandia. This is further substantiated by the population curves depicted in Figure 3 in the paper. There is only one comment on the representation of *O. brumata* in these curves

“ *Curiously, O. brumata displayed a second, much smaller, peak in abundance during the years and sites where A. aurantiaria was most abundant (2005–2009)*”.

In fact, Figure 3 in the paper catches two successive travelling waves, the waves of the 1990s and the 2000s, which together shed light on the “curious” peak. The *O. brumata* population curves for the eastern-most and western-most sites reveal this most clearly. At the eastern-most inland pair of sites (11, 12) and the north-eastern-most island sites (8, 10), the wave of the 1990s had already passed at the time of the study (cf. Ims *et al.* 2004 above ^11^). Instead, the wave of the 2000s had arrived and culminated in 2004–2006 after a distinct low in 2000–2002. Farthest to the west, at sites 1–3 and 5, both waves are represented, i.e., the wave of the 1990s culminating in 2000 and then crashing to a low (cf. Ims *et al.* 2004 above ^11^; see also Tenow *et al.* 2007 ^3^), followed by a weaker peak (= the “curious” peak) that must be identified as the wave of the 2000s. It culminated in 2006–2008, with a delay of 1–2 years since it stood at the eastern sites 11 and 12. Where *A. aurantiaria* occurred (as an associated geometrid), it culminated in about the same years as *O. brumata* at most sites. At the remaining, intermediary sites (4, 6, 7, 9), the crash of the wave of the 1990s remains visible plus the wave of the 2000s. The two waves are distinctly separated by a population low around (2002) 2004–2005 for sites 1–3, 5 (Figure 3 in the paper). Hence, during the study period, the two waves crossed the area in continuous movements from east to west, first the wave of the 1990s, then that of the 2000s. The interval between the peaks was short, about 7–9 years compared with the overall average of 9–10-years; however, short intervals have not been uncommon in northern Fennoscandia ^8, 9^.

In conclusion, as demonstrated with the “curious” *O. brumata* peak as an example, it is not possible to interpret long-term data fully if the continental-scale outbreak waves of *O. brumata* (and associated geometrids) that pass northern Fennoscandia are neglected or not known (however, see Hagen *et al.* 2010 ^18^, referring to Tenow *et al.* 2007 ^3^).
