# Supplementary material for: Discovery of continental-scale travelling waves and lagged synchrony in geometrid moth outbreaks prompt a re-evaluation of mountain birch/geometrid studies
Source: F1000Res. 2013 Sep 6;2:128. Originally published 2013 May 20. [Version 2] doi: 10.12688/f1000research.2-128.v2 (PMC3892914; doi:10.12688/f1000research.2-128.v2)
Supplement: Peer review history

# Peer review: Referee response for version 2

**DOI:** 10.5256/f1000research.2315.r3275

**Published:** 2014-01-22

**Authors:** Patrick Tobin

**Affiliations:** 1 Ecology and Management of Invasive Species and Forest Ecosystems Unit, USDA Forest Service, Northern Research Station, Morgantown, WV, USA

I have read this submission. I believe that I have an appropriate level of expertise to confirm that it is of an acceptable scientific standard.

# Peer review: Referee response for version 2

**DOI:** 10.5256/f1000research.2315.r3274

**Published:** 2014-01-22

**Authors:** Judith H Myers

**Affiliations:** 1 Department of Zoology, and Biodiversity Research Centre, University of British Columbia, Vancouver, BC, Canada

I have read this submission. I believe that I have an appropriate level of expertise to confirm that it is of an acceptable scientific standard.

# Peer review: Referee response for version 1

**DOI:** 10.5256/f1000research.855.r967

**Published:** 2013-05-28

**Authors:** Patrick Tobin

**Affiliations:** 1 Ecology and Management of Invasive Species and Forest Ecosystems Unit, USDA Forest Service, Northern Research Station, Morgantown, WV, USA

The study of forest insect population dynamics has a long and storied history, and many forest insect species have served as model systems for studying conceptual processes of population biology and ecology. This is particularly the case for two geometrid species undergoing cyclical populations in Fennoscandia. A recent paper by Tenow *et al.* (2013) ^1^, recommended in F1000Prime ^2^) revealed the presence of regular travelling waves for the winter moth ( *Operophtera brumata*) across the European continent, providing evidence of the importance of continental processes in understanding regional and local dynamics of cyclical populations.

In this commentary paper, Tenow contends that due to the presence of these continental-scale travelling waves, other studies within this system that do not account for these patterns could be in fact missing an important driver of population dynamics. Tenow critiques five papers to challenge their respective conclusions. 

This commentary should stimulate a thoughtful dialogue by all involved in these studies, which undoubtedly would a benefit to others involved in the study of insect spatial and temporal dynamics.

I have read this submission. I believe that I have an appropriate level of expertise to confirm that it is of an acceptable scientific standard.

## References

[1] TenowONilssenACBylundHet al.: Geometrid outbreak waves travel across Europe.*J. Anim. Ecol.*.2013;82(1) : 12884-9510.1111/j.1365-2656.2012.02023.x22897224

[2] F1000Prime Recommendations, Dissents and Comments for [Tenow O et al., J Anim Ecol 2012.]. *F1000Prime*.24 May 2012; 12810.3410/f.717954042.793459722

# Peer review: Referee response for version 1

**DOI:** 10.5256/f1000research.855.r965

**Published:** 2013-05-24

**Authors:** Judith H Myers

**Affiliations:** 1 Department of Zoology, and Biodiversity Research Centre, University of British Columbia, Vancouver, BC, Canada

Population cycles of two species of forest caterpillars, winter moth *Operophtera brumata* and the autumnal moth *Epirrita autumnata* have been studied extensively over both spatial and temporal scales in Norway, Sweden and Finland by a number of workers. Two major findings are that the outbreaks of winter moth tend to lag those of autumnal moth by one to two years, and waves of population outbreaks of both species occur from east to west across Fennoscandia with a ten-year periodicity ( Tenow 2013). Over the years there has been much interest in the synchronous population dynamics of cyclic species and whether this is associated with some environmental cue, wide ranging dispersal, or the movement of natural enemies among populations. The lag in outbreaks of winter moth and autumnal moth and the waves of outbreaks suggest that a single environmental cue is unlikely to explain the outbreak dynamics.

Here Tenow reinterprets the results of 5 publications of population outbreaks of winter moth and autumnal moth in Norway to show how interpretations could be different if the lag between the two species and the waves of outbreaks were considered. The descriptions are complex and the paper would be clearer if the figures from the original papers were included so that it was evident what the population trends were that were being discussed. Given the extensive amount of work on this system it is useful to reevaluate earlier studies in light of more recent findings as is done here. 

Remaining interesting questions are 

1. What factors determine the geographical waves of outbreaks? And 

2. Why does this apparently consistent lag occur in outbreaks of two species feeding on the same host trees?

I have read this submission. I believe that I have an appropriate level of expertise to confirm that it is of an acceptable scientific standard.

# Response

**Published:** 2013-09-06

**Authors:** Olle Tenow

**Affiliations:** Swedish University of Agricultural Sciences, Sweden

Professor Meyers, many thanks for your referee report of 24 May! In it you asked for figures from the papers I treated to be inserted in my article to make it easier for the reader to follow the text. I can only say that I asked the copyright-holders for permission to use some specified figures but they, sorry to say, did not allow me to do that.                                                                        
